# Competency Assessment in Senior Emergency Medicine Residents for Core Ultrasound Skills

**DOI:** 10.5811/westjem.2015.9.28587

**Published:** 2015-11-12

**Authors:** Jessica N. Schmidt, John Kendall, Courtney Smalley

**Affiliations:** * University of Wisconsin School of Medicine and Public Health, Department of Emergency Medicine, Madison, Wisconsin; † Denver Health Medical Center, Department of Emergency Medicine, Denver, Colorado; ‡ University of Colorado School of Medicine, Department of Emergency Medicine, Denver, Colorado

## Abstract

**Introduction:**

Quality resident education in point-of-care ultrasound (POC US) is becoming increasingly important in emergency medicine (EM); however, the best methods to evaluate competency in graduating residents has not been established. We sought to design and implement a rigorous assessment of image acquisition and interpretation in POC US in a cohort of graduating residents at our institution.

**Methods:**

We evaluated nine senior residents in both image acquisition and image interpretation for five core US skills (focused assessment with sonography for trauma (FAST), aorta, echocardiogram (ECHO), pelvic, central line placement). Image acquisition, using an observed clinical skills exam (OSCE) directed assessment with a standardized patient model. Image interpretation was measured with a multiple-choice exam including normal and pathologic images.

**Results:**

Residents performed well on image acquisition for core skills with an average score of 85.7% for core skills and 74% including advanced skills (ovaries, advanced ECHO, advanced aorta). Residents scored well but slightly lower on image interpretation with an average score of 76%.

**Conclusion:**

Senior residents performed well on core POC US skills as evaluated with a rigorous assessment tool. This tool may be developed further for other EM programs to use for graduating resident evaluation.

## INTRODUCTION

Quality resident education in point-of-care ultrasound (POC US) has become increasingly important in the practice of emergency medicine (EM). Several guidelines have been proposed for US curriculum and training.^1^,^2^ Most recently, in 2008 the Council of EM Residency Directors (CORD) together with leaders in the field of POC US published guidelines for minimum education standards. This consensus group made recommendations regarding methods for competency assessment, including a practical examination with direct assessment of US skills and an assessment of image interpretation.^3^

Previous research has shown that there are reliable methods to assess US skills in trainees including using observed clinical skills exam (OSCE),^4^–^8^ written image interpretation,^9^,^10^ and protected hands-on training.^11^ However, despite validation of these methods, it remains unclear how best to evaluate residents in POC US. In 2013, Amini et al published a survey study reporting wide variation in current EM residency practices in competency assessment demonstrating that 21% used standardized direct observation tools (SDOTS), a third used multiple choice questions, and another third administered practical exams.^12^

Despite the development of minimum education standards for POC US, it is unclear whether residents are graduating with the required skill set. We sought to create a rigorous assessment tool using previously validated methods including an OSCE and written exam to evaluate both image acquisition and image interpretation in residents nearing graduation. The aim of our study was to assess how such an assessment tool could aid in evaluating senior residents in five core US skills as defined by the 2008 CORD document. This is, to our knowledge, the first study since publication of the CORD recommendations to describe a methodology for senior resident assessment in POC US.

## METHODS

We conducted the study at an urban academic emergency department with an annual census of 55,000. Nine senior residents in a four-year accreditation council for graduate medical education accredited EM residency program participated in the study. Participation was voluntary and the study was approved by the institutional review board with written consent obtained from participants.

All residents completed a two-week US rotation during their first year, acquired a minimum of 150 scans and participated in didactics throughout residency, in addition to using POC US during their clinical experience. One resident also completed a two-week elective in US. On average, residents had spent 37 months in residency at the time of evaluation.

We evaluated residents in US image acquisition and image interpretation for five core EM US applications including echocardiogram (ECHO), aorta, focused assessment with sonography for trauma (FAST), pelvic (trans-abdominal and trans-vaginal) and central line placement.

### Image Acquisition

To assess image acquisition, residents were asked to perform five basic POC US skills on a live standardized patient model while two independent US-trained EM physicians completed a pre-defined OSCE checklist regarding their performance. Checklists for ECHO, aorta, FAST and pelvic exams were created by the Academy of Emergency US and are published in the list of CORD Assessment Methods.^13^ We obtained the checklist for central line placement from a previously published checklist.^14^ Table 1 shows the itemized components of each checklist scored as one point each. As each resident was scored by two examiners, there was a total possible score of 126 points. Results were coded so that the authors of this paper were blinded to the individual reviewer and the resident participant. Residents were given 45 minutes to complete all exams.

### Image Interpretation

To assess image interpretation, residents were asked to complete a computer-based multiple-choice quiz. This quiz contained live cine-clips and still images with normal and abnormal pathology. The question bank was created by www.emsono.com. Table 2 outlines the concepts tested and the breakdown of normal and abnormal pathology. Results of the quiz were compiled by an external server and presented in a de-identified data set to the study authors.

## RESULTS

### Image Acquisition

For the image acquisition, the average total score from the OSCE checklist was 92 out of 126 (74%) with a range from 75 to 110 (61–87%). When excluding advanced US competencies including evaluation of ovaries, advanced ECHO, and aortic root measurement, the average score was 85.7 out of 100 (85.7%).

Central line/FAST*:* Residents scored highest in the central line application with all residents scoring 100% correct. They also scored highly in the FAST exam with an average of score of 21.3 out of 22 (97%)

Aorta: On assessment of the abdominal aorta, residents scored an average of 17.7 out of 20 points (88%). On advanced aortic imaging, however, only three residents correctly identified the superior mesenteric artery and/or the celiac trunk and one resident identified the spinal stripe.

Pelvic: For the trans-abdominal and trans-vaginal pelvic assessments, the average scores were 10 out of 12 (85%) and 9.6 out of 12.0 (80%) respectively. The most frequently missed structures on trans-abdominal US were the left and right ovaries (scored one point each). For the trans-vaginal US, in addition to missed ovaries, two of the residents reversed the coronal and sagittal orientations.

ECHO: For ECHO evaluation, resident averaged a score of 18.7 out of 30 (62%). Two residents had outlying low scores of 4 and 9 due to inability to obtain an apical four chamber and parasternal short axis views. Six residents incorrectly identified or incorrectly measured the aortic root and four incorrectly identified the chambers on parasternal long-axis view.

### Image Interpretation

For image interpretation, the average score was 76% with a range from 68 to 89%. One resident data point was excluded due to an incomplete on-line quiz due to technical errors. The majority of the scores ranged from 71–79 percent and only one score was below 70%.

## DISCUSSION

Our study demonstrates that senior residents performed well at image acquisition in several applications including central line placement, FAST, and basic aorta. There was more variation in our cohort in pelvic and advanced ECHO and aorta. For pelvic image acquisition, residents primarily had difficulty identifying the ovaries, and for ECHO there was variation in ability to obtain parasternal-short and apical four chamber views. For aorta, there was difficulty with the superior mesenteric artery and spinal stripe. Adnexal pathology, heart chamber size and comparison, and aortic anatomy are all considered “advanced skills” by CORD. Because the OSCE included both “core” and “advanced” skills as defined by CORD, the limitations noted by our resident cohort may not necessarily mean they are not meeting “core skills,” but may reflect deficits in more advanced POC US skills. When the data was re-analyzed excluding those aspects of the exams that were considered advanced skills, overall scores went from 74 to 85.7%. For future studies, researchers may want to consider an edited version of the CORD OSCE that includes only core skills.

Overall, there was a tendency for residents to score higher on the image acquisition than image interpretation. This may be due to the method of scoring for the two different testing modalities. Additionally, residents have been exposed to hands-on US during their clinical training, primarily identifying normal structures. It may be that more exposure to pathology, as tested in the image interpretation section, is required.

Although our residents performed well on testing overall, a score that reflects “competency” has yet to be defined. Leaders in the field of POC US have suggested that a comprehensive approach is needed to fully assess an individual resident’s competency in POC US.^3^,^15^ Although our study offers a rigorous tool using validated methods, further studies are warranted to evaluate how performance on these measures correlates to clinical performance.

## LIMITATIONS

One potential limitation was possible reviewer bias. Due to the nature of the OSCE, the two reviewers were not blinded to the residents and may have had previous clinical experience with them, which may have skewed their interpretation of image acquisition. To best compensate for this limitation, two reviewers were selected instead of one to ensure that there was consistency. Future studies may use independent US-trained reviewers to limit this bias.

Additionally, this study was performed on a small cohort of residents at a single institution. While we were able to identify areas of weakness overall for our program and for individual residents, it remains to be seen how a similar assessment would work at other institutions.

Finally, with the OSCE exams put forth by CORD, there is no recommendation for how many items on the checklist indicate “competency” or how these scores correlate with clinical performance. Reproducing this study across institutions may allow educators to define an acceptable score for competency.

## CONCLUSION

This is the first paper to measure POC US skills in senior residents using a rigorous methodology to assess both image acquisition and image interpretation in core EM applications as defined by CORD. Overall, we found that senior residents performed well on image interpretation but had difficulty with image acquisition in more advanced US applications. Further work by other institutions and leaders in US is needed to translate performance on these core measures to overall clinical performance.

## Figures and Tables

**Table 1:** Point-of-care ultrasound exam type and area of evaluation scored as one point each for objective structured clinical examination.

Exam type	Area of evaluation
ECHO (15 points)	Correct transducer selection
	Identifies parasternal long axis view with RV, LA, LV
	Measures aortic outflow tract
	Identifies parasternal short axis view with RV, LV
	Identifies apical four-chamber view with RV, LV, RA, LA
Aorta (18 points)	Correct transducer selection
	Identifies target and inferior vena cava by compression and/or doppler
	Obtains trans. proximal, middle, distal and bifurcation views
	Obtains longitudinal aorta view
	Performs correct measurement of aorta
	Identifies vertebral body
FAST (11 points)	Correct transducer selection
	Subxiphoid view
	Identifies right upper quadrant with Morisons, tip of liver, inf. pole kidney
	Identifies left upper quadrant view
	Identifies the splenorenal recess
	Visualizes the inferior pole of the left kidney
	Identifies pelvic view in transverse and sagittal planes
Pelvic (12 points)	Correct transducer selection
Trans-abdominal	Obtains uterus view in long and short axis
	Scans through bilateral ovaries in two planes
	Explains how to calculate the fetal heart rate with M-mode
Trans-vaginal	Obtains coronal and sagittal uterus view
Central line (7 points)	Correct transducer selection
	Explains probe positioning/marker orientation
	Identifies target and associated artery
	Measures depth of vein
	Appropriately demonstrates needle entry/angle of insertion

*ECHO*, echocardiogram; *RV*, right ventricle; *LA*, left atrium; *LV*, left ventricle; *RA*, right atrium; *FAST*, focused assessment with sonography for trauma

**Table 2:** Question categories for image-interpretation quiz.

Category	Subcategory	Questions (# abnormal)
AAA	N/A	5 (2)
FAST	N/A	5 (2)
ECHO	Pericardial effusion	5 (2)
	RV: LV	5 (2)
	Ejection Fraction	5 (2)
Pelvic	Pregnancy	6 (3)
	Yolk sac/gestational sac/fetal pole	2
	Positive FAST with no IUP and +UPT	2
Central line	Identifies needle tip vs artifact Identifies correct vessel	3
Total		38

*AAA*, abnormal aortic aneurysm; *FAST*, focused assessment with sonography for trauma; *ECHO*, echocardiogram; *RV*, right ventricle; *LV*, left ventricle; *IUP*, intrauterine pregnancy; *UPT*, urinary pregnancy test
